# MSFFAL: Few-Shot Object Detection via Multi-Scale Feature Fusion and Attentive Learning

**DOI:** 10.3390/s23073609

**Published:** 2023-03-30

**Authors:** Tianzhao Zhang, Ruoxi Sun, Yong Wan, Fuping Zhang, Jianming Wei

**Affiliations:** 1 Shanghai Advanced Research Institute, Chinese Academy of Sciences, Shanghai 201210, China; 2 School of Electronic, Electrical and Communication Engineering, University of Chinese Academy of Sciences, Beijing 100049, China; 3 School of Information Science and Technology, ShanghaiTech University, Shanghai 201210, China; 4 State Key Laboratory of Geomechanics and Geotechnical Engineering, Institute of Rock and Soil Mechanics, Chinese Academy of Sciences, Wuhan 430071, China

**Keywords:** few-shot object detection, few-shot learning, attention mechanism, multi-scale feature fusion

## Abstract

Few-shot object detection (FSOD) is proposed to solve the application problem of traditional detectors in scenarios lacking training samples. The meta-learning methods have attracted the researchers’ attention for their excellent generalization performance. They usually select the same class of support features according to the query labels to weight the query features. However, the model cannot possess the ability of active identification only by using the same category support features, and feature selection causes difficulties in the testing process without labels. The single-scale feature of the model also leads to poor performance in small object detection. In addition, the hard samples in the support branch impact the backbone’s representation of the support features, thus impacting the feature weighting process. To overcome these problems, we propose a multi-scale feature fusion and attentive learning (MSFFAL) framework for few-shot object detection. We first design the backbone with multi-scale feature fusion and channel attention mechanism to improve the model’s detection accuracy on small objects and the representation of hard support samples. Based on this, we propose an attention loss to replace the feature weighting module. The loss allows the model to consistently represent the objects of the same category in the two branches and realizes the active recognition of the model. The model no longer depends on query labels to select features when testing, optimizing the model testing process. The experiments show that MSFFAL outperforms the state-of-the-art (SOTA) by 0.7–7.8% on the Pascal VOC and exhibits 1.61 times the result of the baseline model in MS COCO’s small objects detection.

## 1. Introduction

Thanks to the development of large-scale computing devices, deep learning has made rapid progress. As a research branch of deep learning, object detection is widely used in production and life due to its excellent stability, high accuracy, and detection speed. It can realize the localization and classification of the objects and mark them in images in the form of text and bounding boxes. However, object detection algorithms based on deep learning usually need to learn the representation of object features from large-scale labeled data before they can classify and locate objects, which consumes many human and material resources [1,2,3]. Additionally, it is challenging to obtain a large amount of data that can be used for training in some application scenarios, such as rare species detection, industrial defect detection, and so on. Inspired by the cognitive characteristics that humans can recognize a new thing through only a few samples, the researchers believe that the neural network imitates human neurons’ reasoning process, so it should also have similar learning capabilities [4]. Therefore, FSOD comes into being, which is dedicated to using only a few training samples to realize the detection function. The current mainstream FSOD methods can be divided into metric learning-based, data augmentation-based, and meta-learning-based methods.

Metric learning-based methods [5,6,7,8] usually utilize the feature distribution of objects for classification. Li et al. [5] propose a boundary balance condition for the target distribution in the feature space. It can reduce the uncertainty of novel class object representation caused by an excessively large feature distance and the difficulty of novel class feature representation caused by an excessively small feature distance. Sun and Karlinsky et al. [6,7] classified features by comparing the distribution distance between query and support features. However, metric learning heavily relies on the sampling strategy. If the strategy is simple, the model can only learn simple distributions and cannot be applied to complex scenarios; if it is tough, the model will have difficulty or even fail to converge. Augmentation-based methods [9,10] enrich data diversity in limited data through various techniques. Li et al. [10] increase the diversity of data by adding noise and occlusion to the images. This can improve the model’s consistent representation of the same object under different conditions. Zhang et al. [9] employed hallucination networks to generate more object proposals, enriching the training data in disguise. However, it still cannot achieve good results in FSOD with very little training data. Meta-learning-based methods [5,11,12,13,14,15,16] avoid the above problems. The model is usually built with a Siamese network structure [17] and learns to discern the objects in the query images by relying on the information provided by the support images. This mode of continuously adapting to each specific task enables the model to obtain an abstract learning ability, which can easily be generalized on a few training samples.

**Figure 1:**
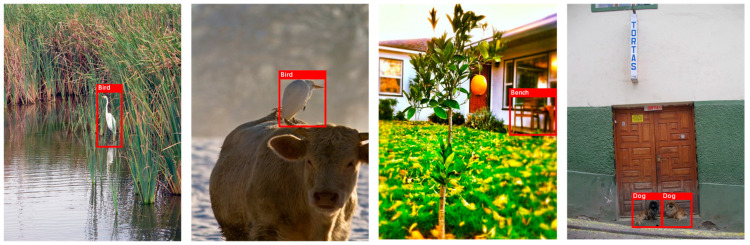
Some support images used for training. The support objects only account for a small proportion in the figure and most of the area is the background, which we call hard samples. It is difficult for the model to extract support features that can represent the desired object category.

In existing meta-learning-based FSOD methods, most models still take ResNet [18] and VGG [19] as the network backbones of the dual-branch structure. This will cause the model to be insufficient in small object detection [20,21], which can only reach 1–3% in mAP (mean average precision) on the MS COCO dataset [22]. At the same time, many hard samples exist in the support branch (as shown in Figure 1). A large percentage of the regions in these samples are background or other category objects, and only a tiny part belongs to useful support objects. This causes the model to fail to obtain the support features that can accurately represent the category, which affects the model’s recognition effect of objects belonging to the same class in the two branches. In addition, during the training process, most models need to filter out the support features of the same class according to the query labels to enhance the query features, highlighting the object features belonging to the same category in the two branches. This seems reasonable, but it only makes use of the support feature information of the same category, and the model does not obtain the ability to actively distinguish the same category of objects from the support images. The model has to manually prepare support images of the same class during the testing process without query labels, making the process more time-consuming and laborious.

To overcome the above deficiencies, this paper proposes the MSFFAL based on meta-learning. First, we adopt a multi-scale feature fusion strategy and design the backbone as ResNet + feature pyramid networks (FPNs) [21] to improve the model’s recognition effect on small objects. Then, we optimize the model’s representation of hard support samples by introducing the channel attention structure SENet [23] in the support branch to weight the features of foreground objects. Finally, we design an attention loss to let query features perform attention calculations with all support features. The computed attention scores constrain the model’s representation of the query features. Through attention loss, the model learns to actively focus on objects of the same category in the two branches and no longer depends on query labels. The experiments on the benchmark datasets Pascal VOC [24,25] and MS COCO [22] prove the effectiveness of our method.

To summarize, the main contributions of this paper are as follows:

(1) We propose an MSFFAL framework for few-shot object detection. The backbone of our model mainly contains the multi-scale feature fusion and channel attention mechanisms. The former is introduced to improve the model’s detection accuracy on the small objects. The latter is adopted to strengthen the model’s representation of hard samples in the support branch and enhance the model attention to foreground object features.
(2) We design an attention loss to enhance the active recognition ability of the model, realize the consistent representation of objects belonging to the same category in the two branches, and improve the model’s generalization ability in novel classes. Based on this, the model no longer relies on the feature selection and avoids model testing difficulty.
(3) We conduct extensive experiments on the benchmark datasets Pascal VOC and MS COCO to verify the effectiveness of our method. The experimental results show that our model is 0.7–7.8% ahead of the SOTAs on the Pascal VOC. We also achieve a substantial lead over the baseline model in MS COCO’s small object detection.

This paper includes five sections: The first is the introduction, which introduces the relevant research background of FSOD and the motivation for our research. The Section 2 presents the related work of FSOD and describes the problems and optimization possibilities of the previous methods. The Section 3 introduces our algorithm in detail. The Section 4 first introduces the dataset selected in this paper and the relevant experimental settings and then shows sufficient experimental results to prove the reliability of our work. The Section 5 summarizes the whole work and concludes.

## 2. Related Works

### 2.1. Object Detection

The object detection algorithm can realize the detection of the targets in the image or video. If there is a target to be detected, it will return the category and bounding box information and mark it in the image. Conventional deep learning-based object detectors can be classified into one- and two-stage detectors. The one-stage detectors directly regress the object bounding boxes and categories through the fully connected layer or the convolutional layer in the deep features, such as the YOLO [26,27,28] series detectors and the SSD [20] detector. These are characterized by a high detection speed but are prone to misjudgment of background information. The two-stage detectors generate the object candidate regions and perform position repair and classification on the candidate regions, such as the faster R-CNN [29,30,31,32] series detectors. Compared with the one-stage detectors, the detection speed of the two-stage detectors is slower, but the detection accuracy is higher than that of the single-stage detectors. Since the region proposal network (RPN) module in faster R-CNN only distinguishes foreground and background information, it has better class independence. This gives faster R-CNN a more significant advantage in generalization to novel classes. Therefore, most current FSOD models take faster R-CNN as their base detector. The method in this paper is also evolved based on this model.

### 2.2. Few-Shot Learning

To allow the deep model to generalize in the target domain with only a few samples, the researchers propose a new machine learning method, namely few-shot learning (FSL) [33,34,35,36,37] for this problem. However, insufficient samples will bring difficulties in model training, resulting in overfitting. Therefore, learning a kind of transferable abstract knowledge so that the deep model can be applied to the target scene with only a little or no training data has become a critical research problem in this field. Early FSL methods mainly focus on classification tasks. At the earliest, Li et al. [4] proposed a method based on the Bayesian framework. They believed computers should learn to use prior knowledge, just like humans can recognize new things from a few examples. Later, Vinyals et al. [38] proposed the matching network to encode images as deeply embedded features and perform weighted nearest neighbor matching to classify query images. Snell et al. [39] proposed a prototype network based on the previous methods, converted the embedded features into feature vectors, and classified samples by measuring the distance between the feature vectors. Recently, Xie et al. [40] found that the few-shot classification accuracy can be improved using Brownian distance instead of the previous Euclidean distance or cosine similarity. The above methods allow the model to no longer focus on the specific category of the object but to learn how to distinguish which objects are in the same category. Therefore, the model also has good generalization performance when facing unseen samples. However, compared with the few-shot classification tasks, FSOD needs to consider both the classification and localization of the objects. Thus, it is more challenging to implement and needs to be the focus of further work.

### 2.3. Few-Shot Object Detection and Meta-Learning Paradigm

FSOD aims to use only a few labeled images to train the model to realize the localization and classification of the objects. Among various FSOD methods, researchers are widely concerned with meta-learning-based models because of their abstract learning ability to better generalize in novel classes. Kang et al. [11] developed a dual-branch detection model based on YOLO. They proposed a reweighting module to realize the weighting of support features to query features, amplifying common object features and enhance the model’s attention to objects belonging to the same category in the two branches. Like the former, Xiao and Yan et al. [14,16] built few-shot detectors based on faster R-CNN, which raises the detection results to a higher level. Fan et al. [12] proposed the attention RPN based on the faster R-CNN. They took support features to perform feature enhancement on query features before feeding them into RPN. This improves the proposal effect of RPN for unseen novel class objects. Zhang et al. [13] generated a feature convolutional kernel for the support branch features and then performed convolution operations on the query features to enhance the object features belonging to the same category. All the above methods focus on enhancing the support features to the query features, ignoring the inherent defects of the model itself. Firstly, the hard samples in the support branch lead to the model’s imprecise representation of support features, affecting the effect of weighting query features. Secondly, the single-level feature maps lead to the model’s poor performance in small object detection. Finally, weighting query features only through the support features of the same category cannot endow the model with the ability to actively identify objects of the same category. To this end, we propose the MSFFAL to overcome the previous shortcomings from the above three perspectives and verify the method’s effectiveness through sufficient experiments.

## 3. Method

Our method has been further innovated and optimized based on the meta R-CNN [14]. We first improved the model’s recognition performance for small objects by introducing a multi-scale mechanism in the feature extraction backbone. Then, we add a channel attention mechanism based on FPN to optimize the model’s representation of hard samples in the support branch and improve detection precision. Finally, we designed an attention loss to let the model learn consistent representations of objects in the two branches of the same category. The model learns to actively identify objects from support samples, leading to an overall improvement in detection performance. In this section, we first make a method definition for FSOD. Then, we introduce the overall architecture of MSFFAL and describe the modules and structures in detail.

### 3.1. Problem Definition

We follow the dataset setup, training strategy, and evaluation methods in [11,14]. We divide the dataset into $C_{b}$ and $C_{n}$, where $C_{b}$ is the base class data with thousands of annotations per class and $C_{n}$ is the novel class data with only one to dozens of annotations per class. The base class and the novel class data do not contain the same object categories, that is, $C_{b}\cap C_{n}=\Phi .$ We first train the model on base classes $C_{b}$ and then fine-tune it on the balance set of $C_{b}$ and $C_{n}$ with only K annotations per class. K is set to different values according to the evaluation indicators of different datasets. For a given N-way K-shot learning task, in each iteration, the model samples a query image and NK support images with N categories and K objects in each category from the prepared dataset as input. Then, the model outputs the detection results of the objects in the query image. Finally, we evaluate the model’s performance by the mAP on the novel classes in the test set.

### 3.2. Model Architecture

**Figure 2:**
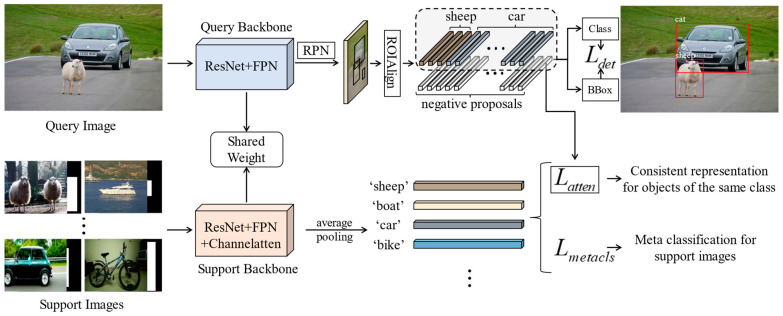
The pipeline of the MSFFAL. The input of the model is a task sample consisting of a query image and a set of support images. The model learns from the input task to discover the objects belonging to the same category in the two branches so as to achieve generalization in the novel classes.

We choose the meta R-CNN, whose backbone is faster R-CNN, as our baseline. The model architecture is shown in Figure 2, which is a Siamese network structure. The upper side of the network is the query branch, which inputs the query image to be detected, while the lower side is the support branch, which inputs the support image-mask pairs for auxiliary detection. We remove the meta learner module in meta R-CNN and realize the information interaction in the two branches through our attention loss. Compared with the baseline, we optimize the backbone of the query and support branches into ResNet + FPN and ResNet + FPN + SENet structures, respectively. The two backbones share weight parameters during the training stage. The query features are passed through RPN and ROIAlign to obtain positive and negative proposal feature vectors. The support features are directly average pooled to obtain support feature vectors representing each support object category. Then, they are used to construct $L_{\mathrm{metacls}}$ to classify support objects and to make attention loss with query positive proposal vectors. The model is trained with three losses, namely:

(1) $$L=\lambda_{1}L_{\det}+\lambda_{2}L_{\mathrm{metacls}}+\lambda_{3}L_{\mathrm{atten}},$$

where $L_{\det}$ is the detection loss of faster R-CNN, $L_{\mathrm{metacls}}$ is the meta-classification loss in the support branch, $L_{\mathrm{atten}}$ is our attention loss, and λ is the weight parameter of the loss.

### 3.3. FPN and SENet

To improve the detection precision of the FSOD model for small objects and the representation effect for hard support samples, we design the feature extraction backbone as an FPN+SENet structure.

**Figure 3:**
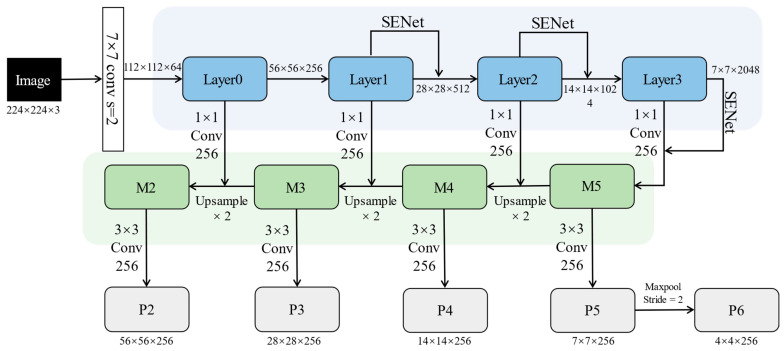
The design of the combination of FPN and SENet. The model achieves the self-attention enhancement of features while realizing multi-scale feature fusion.

As shown in Figure 3, FPN mainly includes a bottom–up line (blue box), a top–down line (green box), and lateral connections (1 × 1 conv 256). Bottom–up is the forward process of the ResNet network. Each layer down-samples the feature maps’ length and width and increases the number of channels. Suppose that the input image size is 224 × 224 × 3, Layer0–Layer3 output feature maps sizes of 56 × 56 × 256, 28 × 28 × 512, 14 × 14 × 1024, and 7 × 7 × 2048, respectively. Top–down is the process of up-sampling the width and height of the feature maps by two times. FPN combines high-level and low-level features through lateral connections to obtain M2–M5 features with 256 channels. Finally, the 3 × 3 convolution kernel is used to convolve the fusion features to eliminate the aliasing effect of up–sampling and get P2–P5 features. P6 is the feature map obtained by P5 after max-pooling with stride = 2. Each level features output by FPN are fed to the RPN module for region proposal. Among them, the low-level features will contribute more proposals for small objects to improve the detection effect of small objects.

**Figure 4:**
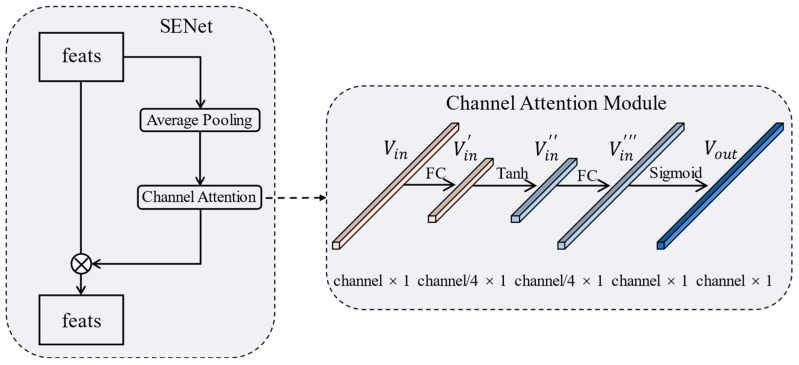
The architecture of SENet. The feature realizes self-attention enhancement through the channel score generated by the channel attention module in the skip connection.

We add the SENet structure based on FPN. The model achieves a channel-level self-attention enhancement through this structure. During the training process, the model continuously learns to improve the representation of hard support samples. The design of SENet is shown in Figure 4. This module adds a skip connection to the output feature layer of the ResNet forward network. In the connection, the feature maps are first average pooled. Then, the channel attention scores are obtained through the channel attention module. Finally, the original feature maps are weighted at the channel level through the scores. The internal structure of the channel attention module is shown on the right side of Figure 4. The input feature vector $V_{\mathrm{in}}\in R^{\mathrm{channel}\times 1}$ is first dimensionally decreased through the first fully connected (FC) layer with a reduction rate of 4 to obtain $V_{\mathrm{in}}^{\prime }\in R^{\mathrm{channel}/4\times 1}$. Then, $V_{\mathrm{in}}^{\prime }$ is followed by the first activation function Tanh to obtain $V_{\mathrm{in}}^{\prime \prime }\in R^{\mathrm{channel}/4\times 1}$. Then, the dimension of $V_{\mathrm{in}}^{\prime \prime }$ is increased through the second FC layer to obtain $V_{\mathrm{in}}^{\prime \prime \prime }\in R^{\mathrm{channel}\times 1}$. Finally, $V_{\mathrm{in}}^{\prime \prime \prime }$ is followed by the second activation function sigmoid to obtain the weight score vector $V_{\mathrm{out}}\in R^{\mathrm{channel}\times 1}$.The whole process can be summarized as:

(2) $$V_{\mathrm{out}}=\mathrm{Sigmoid}({FC}_{2}(\mathrm{Tanh}({FC}_{1}(V_{\mathrm{in}}))))$$

Two different activation functions are used to increase the network’s nonlinearity and enrich the network’s expressive ability. SENet allows the support branch to output high-quality support feature vectors for meta-classification and the construction of attention loss, improving the model detection performance.

### 3.4. Attention Loss

Meta learner is the core module in meta R-CNN, which uses the same category support features to weight the query features. This weighting method causes the model to lack the ability to actively identify objects of the same category, and the dependence on the query labels to select the support features makes model testing difficult. To remedy these, we design an attention loss to replace the meta learner module in the baseline model meta R-CNN.

**Figure 5:**
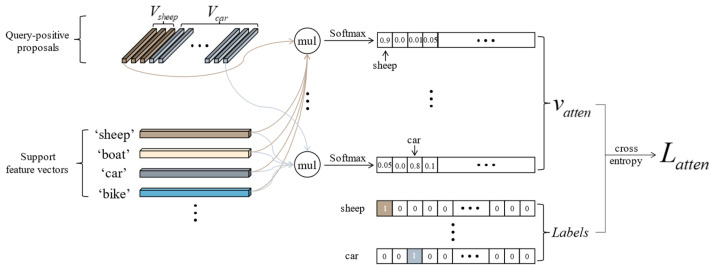
The diagram of the attention loss. It is constructed by the query-positive proposals and all support features.

The essence of the attention loss lies in utilizing the support features to establish a mapping between query-positive proposal features and their corresponding categories. Through training, a strong response is generated between objects of the same category in two branches. The model learns to recognize objects of the same category in two branches while also discriminating objects of different categories. As shown in Figure 5, we extract all query-positive proposal feature vectors $V_{\mathrm{sheep}},V_{\mathrm{car}}\in R^{256\times 1}$ according to the intersection over union (IOU) between the predicted bounding boxes generated by the RPN and the ground truth. We then perform a matrix multiplication operation between all positive proposal feature vectors and the transpose of the support feature vectors $V_{\mathrm{support}}^{T}\in R^{1\times 256}$ and put them through softmax to obtain attention vectors $V_{\mathrm{atten}}\in R^{N}$, where N denotes the number of input support images in each iteration. Each element in $V_{\mathrm{atten}}$ represents a support category. Suppose the category of the positive proposal is consistent with that of the support vector. In that case, we expect the value of the element position corresponding to this category to be close to 1; otherwise, it is close to 0. To achieve the goal above, we concatenate all the attention vectors $V_{\mathrm{atten}}$ together to obtain the score matrix $M_{\mathrm{score}}\in R^{\mathrm{Np}\times N}$, where Np is the numbers of positive proposals, and Labels corresponds to each positive proposal to constrain the trend of $M_{\mathrm{score}}$, that is, the proposed attention loss:

(3) $$L_{\mathrm{atten}}=-M_{\mathrm{score}}\log(M_{\mathrm{labels}})$$

where $M_{\mathrm{labels}}\in R^{\mathrm{Np}\times N}$ represents the concatenation of the Labels.

Through the attention loss, on the one hand, the model can learn a consistent representation of objects belonging to the same category in the two branches during the training process. On the other hand, the model learns an abstract and easily transferable meta-knowledge in this way. Thus, it can also show an excellent generalization performance when facing unseen novel class objects. 

## 4. Experiments

### 4.1. Setup

#### 4.1.1. Datasets and Preparation

We validate our method on two benchmark object detection datasets, Pascal VOC and MS COCO. The few-shot object detection datasets are constructed by splitting the above two datasets.

**Pascal VOC:** The Pascal VOC dataset contains 20 object categories in total. The dataset is divided into a base set and a novel set by three splits. The base set of each split includes 15 categories, and the novel set contains 5 categories. The novel sets of each split are: novel set 1: {“bird”, “bus”, “cow”, “motorbike”, “sofa”}; novel set 2: {“aircraft”, “bottle”, “cow”, “horse”, “sofa”}; novel set 3: {“boat”, “cat”, “motorbike”, “sheep”, “sofa”}. Novel set 2 and 3 are more challenging to train than novel set 1. We call them hard samples. The model is trained under the condition that only 1, 2, 3, 5, and 10 novel class samples are provided. The performance of the 1-, 2-, 3-, 5-, and 10-shot fine-tuning models is evaluated by the mAP in the test set.
**MS COCO:** There are 80 object categories in the MS COCO dataset split into a base set containing 60 categories and a novel set having 20 categories. The model is trained on the MS COCO dataset under the condition that only 10 and 30 novel class samples are provided. The performance of the 10- and 30-shot fine-tuning models is evaluated by the mAP in the test set.

#### 4.1.2. Implementation Details

Firstly, we pre-train our feature extraction module on the large-scale dataset ImageNet [41] and then train and fine-tune our model end-to-end on the Pascal VOC and MS COCO. We use two pieces of NVIDIA RTX 3090 24 g for model training. We choose stochastic gradient descent (SGD) as the training optimizer with momentum and decay set to 0.9 and 0.0001, respectively. On the Pascal VOC dataset, we performed 18,000 iterations with a learning rate of 0.001 in the first 16,000 iterations and 0.0001 in the last 2000 iterations during the base classes training phase. In the novel classes fine-tuning stage, 300, 600, 900, 1200, and 1500 iterations with a learning rate of 0.001 were performed for the 1-, 2-, 3-, 5-, and 10-shot settings, respectively. On the MS COCO dataset, we performed 120,000 iterations with an initial learning rate of 0.005 in the first 110,000 iterations and 0.0005 in the last 10,000 iterations during the base classes training phase. In the novel classes fine-tuning stage, 5000 and 8000 iterations with a learning rate of 0.001 were performed for the 10- and 30-shot settings, respectively.

### 4.2. Comparison with the State-of-the-Arts

In this section, we compare our model with the popular approaches in recent years on the Pascal VOC and MS COCO datasets. On the Pascal VOC dataset, we only compare the mAP obtained by the models in the novel classes. The evaluation metrics on the MS COCO dataset are more abundant, mainly comparing the model’s AP_50__–__95_, AP_50_, AP_S_, AP_M_, and AP_L_.

#### 4.2.1. Performance in the Three Novel Sets of the Pascal VOC

The detection results of our model in the three novel sets of the Pascal VOC are shown in Table 1, Table 2 and Table 3. In the tables, “shot” refers to the number of annotations provided during model training; “1-, 2-, 3-, 5-, and 10-shot” refer to the mAP of the model’s performance on novel class object detection in the test set, trained with only 1, 2, 3, 5, and 10 annotations provided per class, respectively. “Mean” denotes the average mAP across the five aforementioned scenarios. We compare and analyze the results with the SOTA methods in recent years, including metric learning models, data augmentation models, and meta-learning models. As shown in Table 1, our model leads the SOTA by 2.5%, 8.2%, 7.8%, and 2.6% in 1-, 2-, 3-, and 5-shot fine-tuning in novel set 1, respectively. As illustrated in Table 2, we outperform all metrics by 1.4%, 2.1%, 4.3%, 6.3%, and 0.7% in novel set 2, respectively. In Table 3, we can find that our model outperforms 5-shot fine-tuning by 1.3% in novel set 3. We also lead the SOTA by 5.8% and 3.9% in the average of all fine-tuning results in novel sets 1 and 2, respectively.

The results in Pascal VOC prove the effectiveness of our method. Our model achieves the best results among the 12 detection metrics and ranks second by a slight margin in the rest of the metrics. Even in the hard sample novel set 2, the model still performs well and obtains a comprehensive lead. Although only one result of the model is leading in Novel set 3, other metrics are close behind, which is still a vast improvement compared to the baseline model. It proves that the channel attention mechanism improves the model’s representation of hard support samples and its detection effect. The addition of the attention loss enables the model to consistently represent the same category of objects in the two branches, enhancing the model’s generalization ability in the novel classes.

#### 4.2.2. Detailed Performance on the Novel Set 1

This section compares the model’s average precision (AP) in the 3- and 10-shot fine-tuning of each novel class in novel set 1 and the mean average precision (mAP) in the novel and the base set. Here, “shot” refers to the number of annotations provided during model training.

It can be observed from Table 4 that our model achieves the maximum detection precision in most of the novel class objects, in which the AP of the “bus” and “cow” even surpasses the detection of the base class objects. However, our model lags behind previous methods in the mAP of the base classes. For this reason, we analyze that more information in the base class is retained because models such as TFA [8] and MPSR [43] inherit the ideas of transfer learning. Thus, they behave very differently in base and novel classes. While attention loss focuses on making the model actively discover the same class of objects in the two branches, its performance on the novel and base classes is relatively balanced, and there will be no massive gap when the detection category changes. In addition, since the base class data can be obtained from open source datasets, FSOD should pay more attention to the model’s performance in the novel class data.

#### 4.2.3. Performance on the MS COCO Dataset

This section displays the detection results of our model on the MS COCO dataset. In Table 5, AP_50–95_ represents the average mAP of the model, with an IOU ranging from 0.5 to 0.95. AP_50_ represents the mAP of the model when IOU = 0.5. AP_S_, AP_M_, and AP_L_ are the mAP of the model for small (area < 32 × 32), middle (32 × 32 < area < 96 × 96), and large (area > 96 × 96) object detection, respectively. Here, “shot” refers to the number of annotations provided during model training. Table 5 shows that, in 10-shot fine-tuning, MSFFAL outperforms previous works by 1.1% in AP_S_. In 30-shot fine-tuning, it leads by 1.4% and 1.3% in AP_50_ and AP_S_, respectively. Additionally, the results in the remaining indicators are close to the previous model. In particular, we can find that our model outperforms the baseline model Meta R-CNN in all metrics with a large margin, especially for small objects. This performance shows that the backbone combines the FPN structure to realize multi-scale feature fusion, providing more levels of features for RPN. Among them, the low-level features offer the model a higher number of small object proposals, thereby effectively improving its detection precision on small objects. In addition, SENet’s optimization of support features and the active identification ability of the model endowed by the attention loss promote the improvement of detection results. However, according to the overall detection results, it can be seen that the performance of MSFFAL on the MS COCO is not as good as that on the Pascal VOC and falls behind previous models in many detection results. Concerning this phenomenon, we consider that the first reason is that the MS COCO dataset is more complex than the Pascal VOC, containing a large number of hard samples and small object samples. Moreover, since support features and query features construct the attention loss, the representation effect of the support features directly affects the final result of the model. Although the corresponding strategy has been adjusted to cover this problem, the model still cannot accurately represent such samples, revealing shortcomings when dealing with complex datasets. This will also be a part of our future work.

#### 4.2.4. Convergence Comparison of Attention Loss on Different Datasets

**Figure 6:**
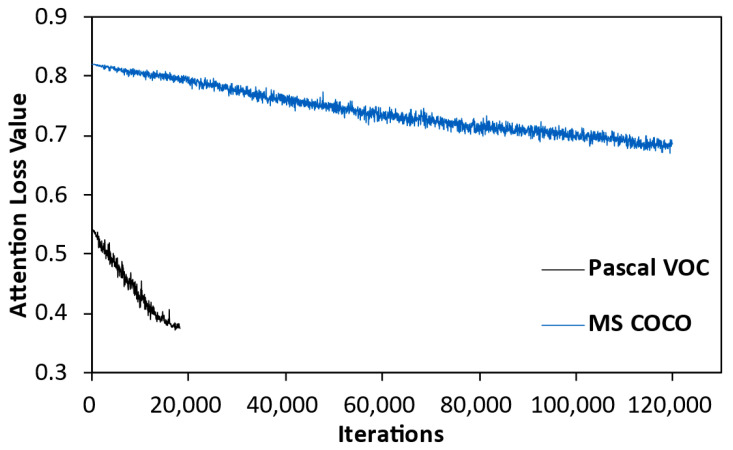
The convergence curves of the changes in attention loss with the number of iterations. The black and blue curves in the figures represent the convergence on the Pascal VOC and MS COCO datasets, respectively.

This section compares the convergence of attention loss during model training on the Pascal VOC and MS COCO datasets. From Figure 6, it can be observed that the attention loss slowly converges in both datasets as the model iterates, demonstrating the effectiveness of the loss. Additionally, we find that compared to the convergence trend on the MS COCO dataset, attention loss converges more quickly and to a greater extent on the Pascal VOC dataset, indicating better convergence performance. This reflects that attention loss is not adept at handling complex datasets such as MS COCO, which has more object categories and contains many small objects. Although the multi-scale feature fusion strategy and attention mechanism somewhat alleviates the impact of this issue, MSFFAL still faces difficulties in representing these objects, which in turn affects the performance of the subsequent attention loss. However, from the overall detection performance of the model, MSFFAL achieves performance improvement compared to the baseline model and surpasses previous algorithms in small object detection.

### 4.3. Ablation Study

In this part, we conducted detailed ablation experiments to verify and analyze the impact of each module on the detection results. We conducted experimental validation on both the Pascal VOC and MS COCO datasets.

#### 4.3.1. The Performance of the Proposed Modules

For a fairer comparison, we performed ablation experiments for the modules on the Pascal VOC hard sample novel set 2 (as shown in Table 6). In the table, FPN represents the multi-scale feature fusion module, and SENet represents the channel attention module. In this part, we compare it with the baseline model meta R-CNN at the same time. It can be observed from the second and third rows of Table 6 that the model’s precision increased by 1.8–24.8% with the addition of attention loss. The fourth row in the table shows that, when we remove the Meta Learner module from the baseline, the model’s precision improves by 0.3–2.8%. Such results reflect that our attention loss plays an essential role in enhancing the model’s active identification ability and generalization effect in the novel classes and dramatically improves the detection precision of the baseline model. For the performance of the model accuracy improvement after removing the meta learner, we analyze that, because the original weighting mechanism selects support features of the same category to weight all positive and negative proposal vectors. This may lead to the enhancement of some negative proposal features, thus impacting the overall recognition performance of the model. In addition, the meta learner relies on the query labels for feature selection which affect the model testing process. For this reason, we directly use attention loss to replace the meta learner. Subsequently, the model’s precision improved with the addition of FPN and SENet. In particular, SENet was essential in enhancing the model training for hard sample tasks. The ablation results in Table 6 prove that the addition of FPN allows RPN to generate more relevant proposals and enhance detection precision. SENet can effectively improve the model’s representation effect on hard support samples. Attention loss enables the model to have an autonomous learning ability, effectively realizes the mining of the same class of objects in the two branches, and improves the detection effect of the few-shot model.

Table 7 compares the contributions of different modules in the detection results on the MS COCO dataset. This section mainly shows the APs and AP_50_ achieved by the model in 10- and 30-shot fine-tuning. It can be seen from Table 7 that although the model faces a more complex dataset, the addition of attention loss still effectively improves the detection accuracy, leading to the AP_50_ of the baseline by 7% and 7.2% in 10- and 30-shot fine-tuning, respectively. In addition, the subsequent combination of SENet and FPN has further improved the detection precision. However, compared to SENet, FPN contributes more to small object detection. Regardless of how the module is combined, adding FPN can promote the detection of small objects by 0.3–0.5%. Finally, compared with the baseline model, MSFFAL achieves a substantial lead.

#### 4.3.2. The Effect of the Position Where the SENet Is Added

In the Section 3 of this paper, we introduce the insertion method of SENet. Since channel-wise attention enhancement is more suitable for deep features with higher semantic levels, there is no way to determine the specific joining position. Thus, in this part, we conduct ablation experiments on the influence of the insertion position of the SENet on the detection results of the Pascal VOC dataset. The experimental results show the average precision of the model for all fine-tuning in the three novel sets (as shown in Table 8). In the table, “Layer0–3” represent the four convolutional layers in ResNet, respectively. It can be seen from Table 8 that, when the channel attention mechanism is added after Layer1, Layer2, and Layer3 of ResNet, the results of the model reach the highest. This also verifies our conjecture that the module does not work with underlying features. In addition, all the experimental results in this paper are based on this setting.

### 4.4. Comparision with the Baseline in Meta Accuracy

Meta accuracy is the classification accuracy of the support branch features. It can reflect the classification effect of the model on the novel classes in the fine-tuning stage.

**Figure 7:**
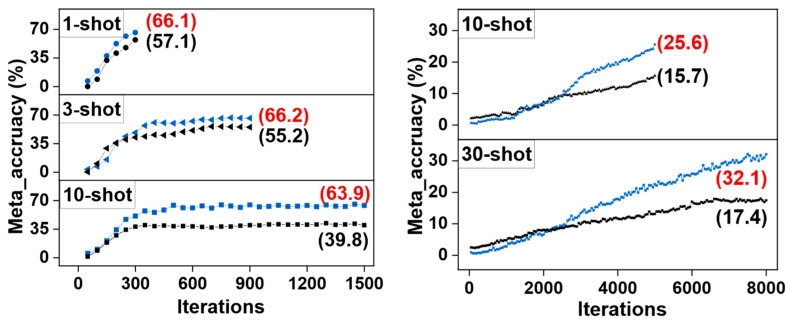
The meta accuracy achieved by the MSFFAL and baseline on the Pascal VOC (**left**) and MS COCO (**right**). The black and blue curves in the figures represent the baseline and MSFFAL, respectively.

As shown in Figure 7, we compare the meta accuracy achieved by our MSFFAL and baseline model Meta R-CNN on the 1-, 3-, and 10-shot fine-tuning on the novel set 1 and 10- and 30-shot fine-tuning on the MS COCO under the same number of iterations. We can observe from the figure that the meta accuracy achieved by MSFFAL exceeds the baseline by 9.0%, 11.0%, 24.1%, 9.9%, and 14.7%, respectively. This proves that MSFFAL can dramatically improve the classification effect of the novel classes and enhance the model’s representation of unseen objects to a large extent. Such results indicate that attention loss has a certain potential in the task of few-shot classification. This will also become our exploration and research direction in the next stage, and performance comparisons will be made with mainstream algorithms such as Brownian distance [40].

### 4.5. Time Complexity Analysis of MSFFAL

Time complexity is an important performance evaluation metric for most deep learning methods and has a significant reference value in deploying and applying models. The time complexity of a model is mainly reflected in the number of floating-point operations. Therefore, we conducted a complexity analysis of MSFFAL, as shown in Table 9. The second column of the table lists the modules included in MSFFAL, which are ResNet50, RPN, Predict head, FPN, and SENet. The third column shows each module’s corresponding floating-point operation counts, measured in giga floating-point operations (GFLOPs). It can be found that the first three modules are the main components of the base detector faster R-CNN and occupy the majority of the model’s computational complexity. The last two modules are introduced in Section 3. The time complexity of attention loss depends on the number of positive proposals, the value is unstable and always small, so it can be ignored. The result shows that introducing FPN increases the model’s time complexity by 2.81 GFLOPs. However, FPN helps the model enhance the detection ability of small objects. Moreover, SENet brings 0.003 GFLOPs to the model but effectively improves the model’s representation and detection of difficult samples. In summary, we can find that improving a model’s performance often comes at the expense of computational complexity. Therefore, reducing this sacrifice is a direction for future exploration.

## 5. Conclusions

We propose the MSFFAL based on meta-learning for few-shot object detection. In addition to optimizing the feature extraction backbone with the FPN structure and SENet, we also designed an attention loss. FPN effectively improves the detection effect of the model on small objects through multi-scale feature fusion. SENet relies on the channel attention mechanism to enhance the representation effect of the support branch on hard samples. Attention loss replaces the weighting module in the baseline model, introduces a mutual constraint between query and support features, and achieves a consistent representation of the objects belonging to the same class. Through this loss, the model learns to actively discover the objects of the same class during the training process and no longer relies on query labels for feature selection. We validate the effectiveness of our method on the benchmark datasets Pascal VOC and MS COCO. In addition to the successful results mentioned above, we identify some limitations in our research. For example, the model’s overall performance in processing complex data still has room for improvement. This is because complex datasets affect the model’s effective representation of features, which in turn affects the effectiveness of the subsequent attention loss. Moreover, there is a general lack of time complexity analysis for FSOD models. We will conduct an in-depth analysis on the complexity issue and research how to reduce the model’s time complexity while maintaining its detection performance. In the future, we will optimize our method to solve the above problems. We will also continue to explore challenging datasets for method validation and performance comparison with representative algorithms.

## Figures and Tables

**Table 1:** Comparison with previous works on novel set 1 of the PASCAL VOC. Conducting 1-, 2-, 3-, 5-, and 10-shot fine-tuning experiments on five novel classes, respectively. The top two results are identified in black and blue bold, respectively.

Method	Novel Set 1					Mean
	1-Shot	2-Shot	3-Shot	5-Shot	10-Shot	
Meta-YOLO [11]	14.8	15.5	26.7	33.9	47.2	27.6
MetaDet [42]	18.9	20.6	30.2	36.8	49.6	31.2
Meta R-CNN [14]	19.9	25.5	35.0	45.7	51.5	35.5
Viewpoint [16]	24.2	35.3	42.5	49.1	57.4	41.7
TFA w/cos [8]	39.8	36.1	44.7	55.7	56.0	46.6
DCNet [15]	33.9	37.4	43.7	51.1	59.6	45.1
AFSOD w/R50 [13]	46.8	**49.2**	50.2	52.0	52.4	50.1
Halluc w/cos [9]	**47.0**	44.9	46.5	54.7	54.7	49.7
TIP [10]	27.7	36.5	43.3	50.2	59.6	43.5
CME w/F R-CNN [5]	41.5	47.5	50.4	58.2	60.9	51.7
FSCE [7]	44.2	43.8	**51.4**	**61.9**	**63.4**	**52.9**
MSFFAL (ours)	**49.5**	**57.4**	**59.2**	**64.5**	**62.9**	**58.7**

**Table 2:** Comparison with previous works on novel set 2 of the PASCAL VOC. Conducting 1-, 2-, 3-, 5-, and 10-shot fine-tuning experiments on five novel classes, respectively. The top two results are identified in black and blue bold, respectively.

Method	Novel Set 2					Mean
	1-Shot	2-Shot	3-Shot	5-Shot	10-Shot	
Meta-YOLO [11]	15.7	15.3	22.7	30.1	40.5	24.9
MetaDet [42]	21.8	23.1	27.8	31.7	43.0	29.5
Meta R-CNN [14]	10.4	19.4	29.6	34.8	45.5	27.9
Viewpoint [16]	21.6	24.6	31.9	37.0	45.7	32.2
TFA w/cos [8]	23.5	26.9	34.1	35.1	39.1	31.7
DCNet [15]	23.2	24.8	30.6	36.7	46.6	32.4
AFSOD w/R50 [13]	**39.4**	**43.1**	**43.6**	44.1	45.7	**43.2**
Halluc w/cos [9]	26.3	31.8	37.4	37.4	41.2	34.8
TIP [10]	22.7	30.1	33.8	40.9	46.9	34.9
CME w/F R-CNN [5]	27.2	30.2	41.4	42.5	46.8	37.6
FSCE [7]	27.3	29.5	43.5	**44.2**	**50.2**	38.9
MSFFAL (ours)	**40.8**	**45.2**	**47.9**	**50.5**	**50.9**	**47.1**

**Table 3:** Comparison with previous works on the Novel set 3 of the PASCAL VOC. Conducting 1-, 2-, 3-, 5-, and 10-shot fine-tuning experiments on five novel classes, respectively. The top two results are identified in black and blue bold, respectively.

Method	Novel Set 3					Mean
	1-Shot	2-Shot	3-Shot	5-Shot	10-Shot	
Meta-YOLO [11]	21.3	25.6	28.4	42.8	45.9	32.8
MetaDet [42]	20.6	23.9	29.4	43.9	44.1	32.4
Meta R-CNN [14]	14.3	18.2	27.5	41.2	48.1	29.9
Viewpoint [16]	21.2	30.0	37.2	43.8	49.6	36.4
TFA w/cos [8]	30.8	34.8	42.8	49.5	49.8	41.5
DCNet [15]	32.3	34.9	39.7	42.6	50.7	40.0
AFSOD w/R50 [13]	**44.1**	**49.8**	**50.5**	52.3	52.8	**49.9**
Halluc w/cos [9]	40.4	42.1	43.3	51.4	49.6	45.4
TIP [10]	21.7	30.6	38.1	44.5	50.9	37.2
CME w/F R-CNN [5]	34.3	39.6	45.1	48.3	51.5	43.8
FSCE [7]	37.2	41.9	47.5	54.6	**58.5**	47.9
MSFFAL (ours)	**40.9**	**45.2**	**50.0**	**55.9**	**56.7**	**49.7**

**Table 4:** Comparison with previous works on each class of novel set 1. The top two results are identified in black and blue bold, respectively. ‘*’ denotes that we reproduce the experimental result. ‘-’ denotes that the result is not recorded.

Shot	Method	Novel Class					mAP	
		Bird	Bus	Cow	mbike	Sofa	Novel	Base
3	Meta YOLO [11]	26.1	19.1	40.7	20.4	27.1	26.7	64.8
	Meta RCNN [14]	30.1	44.6	50.8	38.8	10.7	35.0	64.8
	MPSR [43]	**35.1**	**60.6**	**56.6**	**61.5**	43.4	**51.4**	67.8
	TFA w/cos [8]	-	-	-	-	-	44.7	**79.1**
	TFA w/cos * [8]	21.9	53.8	56.5	54.2	**47.3**	46.7	**78.5**
	MSFFAL (ours)	**57.9**	**67.4**	**64.5**	**58.1**	**48.2**	**59.2**	65.9
10	Meta YOLO [11]	30.0	62.7	43.2	60.6	39.6	47.2	63.6
	Meta RCNN [14]	52.5	55.9	52.7	54.6	41.6	51.5	67.9
	MPSR [43]	48.3	73.7	68.2	**70.8**	**48.2**	**61.8**	**71.8**
	TFA w/cos [8]	-	-	-	-	-	56.0.	78.4
	TFA w/cos * [8]	39.0	71.9	59.9	**70.4**	48.2	57.8	**79.1**
	MSFFAL (ours)	**61.2**	**74.0**	**72.4**	64.6	**42.8**	**62.9**	68.7

**Table 5:** Comparison with previous works on the MS COCO. Conducting 10- and 30-shot fine-tuning experiments on 20 novel classes, respectively. The top two results are identified in black and blue bold, respectively. ‘-’ denotes that the result is not recorded.

Shot	Method	AP_50–95_	AP_50_	AP_S_	AP_M_	AP_L_
10	Meta YOLO [11]	5.6	12.3	0.9	3.5	10.5
	MetaDet [42]	7.1	14.6	1.0	4.1	12.2
	Meta RCNN [14]	8.7	19.1	2.3	7.7	14.0
	TFA w/fc [8]	9.1	17.3	-	-	-
	TFA w/cos [8]	9.1	17.1	-	-	-
	Viewpoint [16]	**12.5**	**27.3**	**2.5**	**13.8**	**19.9**
	MSFFAL (ours)	**11.1**	**26.9**	**3.6**	**11.2**	**18.0**
30	Meta YOLO [11]	9.1	19.0	0.8	4.9	16.8
	MetaDet [42]	11.3	21.7	1.1	6.2	17.3
	Meta RCNN [14]	12.4	25.3	2.8	11.6	19.0
	TFA w/fc [8]	12.0	22.2	-	-	-
	TFA w/cos [8]	12.1	22.0	-	-	-
	Viewpoint [16]	**14.7**	**30.6**	**3.2**	**15.2**	**23.8**
	MSFFAL (ours)	**14.3**	**32.0**	**4.5**	**14.7**	**23.0**

**Table 6:** Study the effect of each module on the detection results of the hard training data novel set 2 of the Pascal VOC dataset. The top results are identified in black bold. ‘✕’ means that the module is not added. ’✓’ means that the module is added.

Model	FPN	SENet	Attention Loss	Novel Set 2				
				1-Shot	2-Shot	3-Shot	5-Shot	10-Shot
Meta R-CNN [14]	✕	✕	✕	10.4	19.4	29.6	34.8	45.4
	✕	✕	✓	35.2	36.9	46.2	45.8	47.2
MSFFAL (ours)	✕	✕	✓	35.5	38.7	47.4	48.6	49.3
	✓	✕	✓	36.1	39.3	45.4	48.3	49.9
	✕	✓	✓	37.6	40.4	46.9	49.0	48.7
	✓	✓	✓	**40.8**	**45.2**	**47.9**	**50.5**	**50.9**

**Table 7:** Study the effect of each module on the model’s detection results in APs and AP50 on the novel class of the MS COCO dataset. The top results are identified in black bold. ‘✕’ means that the module is not added. ’✓’ means that the module is added.

Model	FPN	SENet	Attention Loss	10-Shot		30-Shot	
				AP_S_	AP_50_	AP_S_	AP_50_
Meta R-CNN [14]	✕	✕	✕	2.3	19.1	2.8	25.3
MSFFAL (ours)	✕	✕	✕	2.5	15.5	2.6	21.2
	✓	✕	✕	2.9	16.5	3.0	24.8
	✕	✕	✓	3.0	26.1	3.7	**32.5**
	✓	✕	✓	3.3	26.2	4.2	31.4
	✕	✓	✓	3.1	26.5	4.0	31.8
	✓	✓	✓	**3.6**	**26.9**	**4.5**	32.0

**Table 8:** Evaluate the effect of the SENet’s position added in ResNet on the detection results of the Pascal VOC dataset. The top results are identified in black bold. ‘✕’ means that the module is not added; ’✓’ means that the module is added.

Model	SENet				Mean		
	Layer0	Layer1	Layer2	Layer3	Novel Set 1	Novel Set 2	Novel Set 3
MSFFAL(ours)	✕	✕	✕	✓	56.4	42.6	47.2
	✕	✕	✓	✓	57.6	44.5	48.7
	✕	✓	✓	✓	**58.7**	**47.1**	**49.7**
	✓	✓	✓	✓	58.2	44.7	47.0

**Table 9:** Time complexity analysis of MSFFAL.

Model	Module	GFLOPs
MSFFAL (ours)	ResNet50	3.53
	RPN	2.51
	Predict head	0.003
	FPN	2.81
	SENet	0.003

## Data Availability

Not applicable.
